# Impact of a Mediterranean Diet Supplemented with Extra Virgin Olive Oil on Gut Microbiota in Fibromyalgia: A Randomized Controlled Trial

**DOI:** 10.3390/life16060894

**Published:** 2026-05-26

**Authors:** Elena Durán González, Jorge Antolín Ramírez Tejero, Ismael San Mauro Martín, Ana Terrén Lora, Marta Pérez Sánchez, Rosa Gómez Morano, Claudia Díaz López, Antonio Martínez Lara, Marta Aguilar Díaz, David Cotán Marín

**Affiliations:** 1 Pronacera, Avenida de la Ingeniería 9, Local 34, Parque Empresarial Arte Sacro (PEASS), 41015 Seville, Spain; j.ramirez@pronacera.com (J.A.R.T.); m.perez@sinae.es (M.P.S.); rosagm@sinae.es (R.G.M.); c.diaz@pronacera.com (C.D.L.); a.martinez@pronacera.com (A.M.L.); d.cotan@pronacera.com (D.C.M.); 2 Centros de Investigación en Nutrición y Salud (CINUSA Group), Research Department, Paseo de la Habana 43, 28020 Madrid, Spain; info@grupocinusa.es (I.S.M.M.); ana.terren@grupocinusa.es (A.T.L.); 3 Independent Researcher, 46007 Valencia, Spain; martaguilardiaz@gmail.com

**Keywords:** fibromyalgia, olive oil, oxidation, microbiota, microbiome, bacteria, diet, polyphenols

## Abstract

Fibromyalgia is a chronic syndrome associated with pain, fatigue, and cognitive symptoms, often linked to gut microbiota alterations. The Mediterranean Diet (MD), particularly extra-virgin olive oil (EVOO), has antioxidant and anti-inflammatory properties that may beneficially modulate the microbiota. We conducted a prospective, randomized, double-blind, placebo-controlled clinical trial that included 250 women (206 with fibromyalgia, 44 controls). Participants followed a MD supplemented with EVOO or refined olive oil (placebo) for six months. Microbiota composition was analyzed at four time points (T0–T3) by 16S rRNA sequencing (V3–V4). At baseline, fibromyalgia patients exhibited reduced microbial diversity compared to controls. While global diversity did not change after intervention, specific taxa increased significantly with EVOO, including *Bacteroides fragilis*, *Anaerotruncus colihominis*, *Adlercreutzia equolifaciens*, and butyrate producers such as *Faecalibacterium prausnitzii*, *Roseburia intestinalis*, and *Agathobacter.* These shifts suggest EVOO supplementation might promote anti-inflammatory and metabolic bacteria, suggesting diet as a complementary strategy to modulate gut microbiota in fibromyalgia.

## 1. Introduction

Fibromyalgia (FM) is a chronic syndrome with a marked social and health impact worldwide that, at present, lacks molecular diagnosis and effective treatment [1]. It is mainly characterized by two symptoms: generalized musculoskeletal pain and persistent fatigue, although they are not the only ones, since alterations in sleep quality, cognitive problems and reproductive complications are also common in these patients. Despite its prevalence, estimated at between 2–4% of the world’s population according to the most complete meta-analysis conducted in 2017 by Heidari et al. [2], the etiopathogenesis of the disease remains unknown.

The onset and course of FM symptoms have been associated primarily with genetic and epigenetic, emotional, and environmental factors, as well as alterations in abnormal pain processing in the central nervous system [3]. Often, patients present with additional symptoms such as cognitive problems known as “brain fog” [4], stiffness, anxiety, depression and headache. These ubiquitous symptoms make the diagnosis particularly challenging [5].

In this context, it becomes increasingly important to explore systemic pathophysiological mechanisms that may account for the symptomatic heterogeneity of FM, beyond a purely neurological or musculoskeletal perspective.

On the one hand, gut microbiota modulates gut-brain axis communication affecting in the regulation of chronic pain and fatigue of FM [6,7]. Gut dysbiosis may promote neuroinflammation by activating immune cells in the blood-brain barrier and spinal cord, contributing to central sensitization and maintenance of chronic pain [6]. As the gut microbiota is altered, intestinal permeability increases, allowing the translocation of lipopolysaccharides (LPS) into the bloodstream, triggering a systemic inflammatory response that promotes the activation of microglia and the release of pro-inflammatory cytokines such as TNF-α, IL-1β, and IL-6 [8]. In this regard, it has been observed that people with FM have an altered gut microbial composition, contributing to systemic inflammation and immune system dysfunction [9] and increasing the presence of symptoms such as widespread pain, fatigue, and mood disorders [10]. These effects can contribute to FM symptomatology, affecting sleep regulation, energy, and stress response.

On the other hand, patients with FM and chronic fatigue seem to have altered hypothalamic-pituitary-adrenal axis. It has been observed that certain gut bacteria, such as the genera *Lactobacillus* and *Bifidobacterium*, can modulate the response of this axis by producing GABA and serotonin, decreasing anxiety and improving stress regulation [7]. Likewise, the composition of microbiota can affect neuroplasticity, since intestinal dysbiosis can reduce levels of brain-derived neurotrophic factor (BDNF), a key protein in neuronal plasticity and depression, common symptoms in people with FM. This, added to the lower production of short-chain fatty acids by altered microbiota, favors cognitive dysfunction and mental fatigue in these patients [7].

Recent studies suggest that an anti-inflammatory diet rich in omega-3 fatty acids, antioxidants, and fiber could help relieve muscle pain and fatigue [11]. The MD has shown to be a promising strategy to improve quality of life in patients with FM. The SYNCHRONIZE+ program evaluated the effect of an intervention based on the MD on people with FM and chronic fatigue syndrome. This intervention had a positive impact on body composition and reduced nighttime food intake, which could be linked to improvements in sleep quality and fatigue [12]. Another study showed that a personalized MD improved the perception of pain, fatigue, and anxiety in FM patients after eight weeks of follow-up, while poor food choices and intake of pro-inflammatory foods contributed to the exacerbation of FM symptoms [13]. Specifically, EVOO, the main source of fat in the MD and known for its antioxidant and anti-inflammatory properties and its high content of polyphenols helps reduce oxidative stress and inflammation in the body [14]. Some studies suggest that regular consumption of olive oil may decrease oxidative stress levels, pain intensity, and improve physical function in people with chronic inflammatory diseases [15]. In fact, EVOO appears to decrease DNA oxidation levels, and increase zinc levels in people with FM, improving their functional capacity and psychological state [16]. The effects of some phytochemical compounds and extracts of the olive tree have also been studied [17]. The polyphenol content of EVOO may contribute to modulating oxidative stress and inflammation indeed, key factors in the pathophysiology of FM [18]. Since oxidative stress contributes to pain sensitization in these patients, the consumption of antioxidant-rich foods, such as EVOO, could be an interesting complementary therapeutic strategy [19]. In addition, it has been shown that the polyphenols present in olive oil promote the growth of beneficial bacteria, such as *Bifidobacterium* and *Lactobacillus*, essential for maintaining a balanced microbiota [20]. This positive effect on the gut microbiota may, in turn, influence the reduction of oxidative stress and inflammation present in people with FM [21]. Thus, the inclusion of EVOO in the diet could not only improve the antioxidant profile of the body but also contribute to the modulation of the gut microbiome, which could offer a complementary approach in the treatment of this disease [22].

Thus, the aim of this study is to evaluate how the MD supplemented with EVOO impacts on the gut microbiota of patients with FM compared to controls.

## 2. Materials and Methods

### 2.1. Study Population

**Figure 1:**
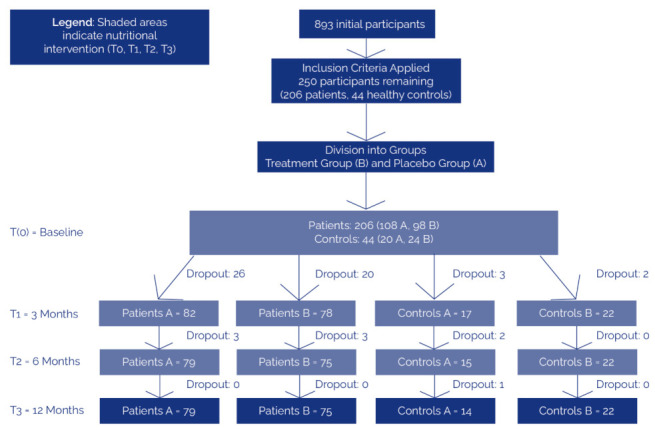
Participant inclusion and dropout diagram.

From the initial group of potential participants, composed of 893 women, a total of 250 participants (206 FM patients and 44 controls) met the inclusion criteria. Specifically, it was necessary not to perform any type of guided or structured physical activity, to follow a balanced diet (according to the principles of the MD), and, in the case of patients with FM, a confirmed diagnosis by a health professional according to the ACR 1990/2010 criteria. The exclusion criteria were as follows: (1) being outside the established age range; (2) having practiced some type of structured or planned physical activity more than 2 times a week during the last month (e.g., group classes, going for a walk, cycling or similar for 30 min or walking more than 10,000 steps/day); (3) having a Body Mass Index (BMI) less than 18.5 or greater than 34.9; (4) suffering from any of the following chronic pathologies: any type of cancer, metabolic diseases (type I and II diabetes, metabolic syndrome), Acquired Immunodeficiency Syndrome (AIDS), inflammatory diseases (rheumatoid arthritis, osteoarthritis), gastrointestinal diseases (Crohn’s disease, ulcerative colitis), cardiovascular diseases (atherosclerosis, cardiomyopathy, stroke), autoimmune diseases (systemic lupus erythematosus, celiac disease, Hashimoto’s thyroiditis, multiple sclerosis); (5) being under intensive pharmacological treatment (3 or more drugs daily) with non-steroidal anti-inflammatory drugs, corticosteroids, analgesics or antidepressants during the month prior to the start of the study; (6) having been under antibiotic treatment during the month prior to the start of the study; (7) consuming alcohol above the limits established in the context of the MD (>12 g/day = 1.5 glasses of red wine); (8) smoking or consuming any type of narcotic substance; (9) lacking a medical report that supports their fibromyalgic condition (exclusively for patients with FM). Figure 1 shows the flow chart of patients throughout the study.

All procedures followed were in accordance with the ethical principles for research with human subjects set out in the Declaration of Helsinki of 1964 and subsequent versions. This study was reviewed and approved by the Ethics Committee for Research in Medicines of the Quirónsalud-Catalunya Hospital Group (Catalonia, Spain), with the reference IDI-20210749, (approval file no. 01/2022). Each participant was verbally informed about the study and signed informed consent prior to undergoing study procedures. The trial registration number (ClinicalTrials.gov) was NCT05921409.

### 2.2. Randomization and Blinding Process

The distribution of participants into one group or another was carried out by randomization by blocks. Being B treated group (EVOO) and A placebo group (refined olive oil), the randomization was applied as follows: ABBA, BAAB, ABAB, BABA, BBAA, AABB; with the aim of ensuring a homogeneous distribution in each group. This is a double-blind study, so both the research team and the participants did not know whether the assigned treatment arm corresponded to the intervention group or the control group.

### 2.3. Sample Size

Intervention studies typically base sample size on effect size and statistical power. However, the exploratory nature of this project and the lack of prior data from interventional studies in FM to estimate effect size supported the use of a prevalence-based approach. The calculation of the cohort size considered the latest and most complete data on the estimated prevalence of FM in Spain published in 2008, which was then around 2.4%. Specifically, this prevalence reached 8.4% in the age range of 40 to 49 years, while in women aged 50 to 59 years it was 6.7% [23]. Following the protocol described by Naing et al., [24] the size needed to obtain a 95% confidence interval (CI) with a α level of 0.05 was estimated. The following formula was used:

$$n=\frac{Z^{2}P(1-P)}{d^{2}}$$

where Z = 1.96 to obtain a statistical confidence of 95%; *p* = 0.0755, expected prevalence in patients between 40 and 59 years of age; and d = 0.0378 as the expected precision value. The result was n = 198, which means that the cohort had to be made up of at least 198 patients with FM. An additional 5% of participants were recruited to account for potential dropouts. Ultimately, this allowed the study to reach the target sample size after considering participant attrition.

### 2.4. Clinical-Nutritional Follow-Up

Four follow-ups were made for data collection: the first one at the beginning, a second one 3 months after the start of the intervention, a third one 6 months after the start of the intervention and coinciding with its end, and a fourth one 12 months after the start of the intervention, after 6 months without intervention. In those visits, participants provided their responses to the clinical questionnaires widely used in FM (SF-36 and FIQR) [25] (Supplementary Material File S1), as well as additional information about their life habits, drug consumption and the appearance of any adverse events.

**Figure 2:**
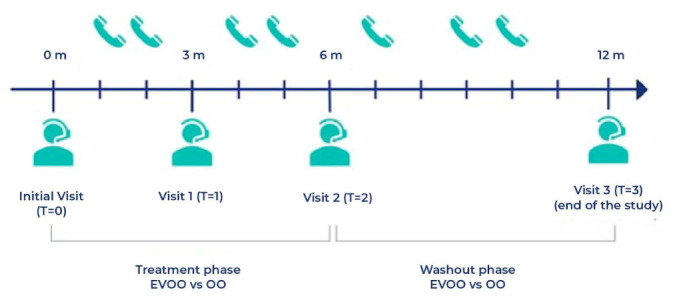
Nutritional follow-up throughout the study.

For better control of the study procedures, a monthly telephone follow-up was performed. These calls were useful to check adherence to treatment, with a 24-h recall questionnaire, to record any changes in drug consumption, to make some modifications to the menus, or to track relevant events that participants reported. The temporal distribution of contacts and calls is shown in Figure 2. All follow-up procedures were conducted by a team of experienced nutritionists.

### 2.5. Dietary Intervention

Each participant received a series of personalized nutritional recommendations and a rotating weekly menu plan, based on the MD pattern (Mediterranean Diet Foundation, www.dietamediterranea.com). Since the selected cohort belonged to a population with a habitual consumption of olive oil, before being included in the study, all participants went through a clearance period prior to the start of the study, consisting of the consumption of 50 mL of placebo (refined olive oil) for 14 days. After this period, participants were randomized. The intervention group (group B) consumed 50 mL of EVOO, and the placebo group (group A) consumed 50 mL of refined olive oil for 6 months. Although its consumption was prioritized raw, distributed in the main meals, to facilitate compliance with the established daily dose of oil, it was allowed, occasionally, to use it in the cooking of food.

### 2.6. Assessment of the Gut Microbiota

For the study of the intestinal microbiota, stool samples were collected by patients in their own homes, with a specific kit for this purpose (DANASTOOL, DANAGEN, Barcelona, Spain), following the instructions of the researchers. From the sample, 2 to 3 small portions of different areas of the stool were taken in the tube provided, which contained a liquid DNA/RNA stabilization solution. After collection, all samples were stored at room temperature (15–25 °C) according to the quality standards set by the manufacturer. In total, each patient collected a total of 4 samples, corresponding to the 4 sampling times.

### 2.7. Fecal Bacterial DNA Extraction

A thermal, chemical, and mechanical lysis protocol was used for fecal DNA extraction with the fecal microbiome DNA kit (DANAGEN, Barcelona, Spain).

DNA quality was assessed using the ThermoFisher Multiskan SkyHigh Microplate Spectrophotometer (Thermo Fisher Scientific, Waltham, MA, USA), a UV/Vis microplate spectrophotometer. A concentration greater than 5 μg/μL and ratios of 260/280 and 260/230 between 1.8–2.1 were considered optimal.

### 2.8. Sequencing of Amplicons of the V3-V4 Regions of 16S rDNA

DNA from stool samples was used to analyze the composition of the gut microbiota. This analysis consisted of the metagenomic sequencing of amplicons of the V3–V4 regions of the 16S ribosomal DNA of bacteria, using the Illumina MiSeq platform at the HelixBioS facilities (HelixBioS, Madrid, Spain). The choice of this methodology facilitated the phylogenetic classification of the sequences [26]. For DNA isolation, library preparation, sequencing and analysis, the workflow recommended by the manufacturer of the sequencing technology used was followed (Illumina, San Diego, CA, USA). The sequencing was performed on fragments with sizes ranging from 2 × 250 to 2 × 300, in paired end format, generating between 150,000 and 200,000 reads per sample.

### 2.9. Taxonomic Assignment

For the taxonomic classification of the amplicons of the 16S gene, the ZOTU (Operational Taxonomic Unit of Zero Radius) was assigned as the unit of work. For this study, a database of the human gut microbiome was used for metagenomics studies based on rRNA 16S (HelixBioS HUMAN GUT 16; version 3.1), owned by Helix Bioinformatics Solutions S.L.

As a result of this analysis, the ZOTU count table was obtained for each sample and each of the sequences of each ZOTU in fasta format.

Once the consensus sequences associated with the detected ZOTUs were obtained, they were aligned against the same taxonomic database, but in two nested rounds with different characteristics, with the aim of achieving the maximum percentage of identification. The first round was a specific identification, in which the sequences were aligned against the taxonomic database developed for the study of the gut microbiota, HUMAN GUT 16S (HelixBios), with a cut-off point of 99% alignment (identity) using the USEARCH-LOCAL algorithm. Once the first alignment was made, with those sequences that were not taxonomically identified in the first round, a generalist alignment was carried out by classifying the ZOTUs using the SINTAX predictor algorithm with the same taxonomic database. A cut-off point based on the identity of this assignment relationship was applied, setting at least 97% identity, so that it was filtered down to the taxonomic level at which this degree of identity was exceeded. For the study of 16S the taxonomic levels studied were kingdom, phylum, class, order, family, genus and species.

### 2.10. Bioinformatic Analysis of Taxonomy

Once the tables of gross abundances in each ZOTU were obtained, the bioinformatic analysis was carried out to establish the differences found between each study variable.

First, the ZOTU tables were unified to work with only those that exceeded 97% identity. After that, all the abundances of those ZOTUS that were annotated for the same specific taxonomic level were unified. In addition, to identify and trace the groups corresponding to the core microbiome, the following conditions were applied: ZOTUs with a minimum ubiquity of 85% in the respective sample group (FM or C) and a minimum abundance of 0.005% in each sample. Once all the filters were applied and the absolute abundance tables were unified in each sample, they were transformed into relative abundances. To do this, the total readings obtained from each sample were calculated, and each of the taxonomic groups identified were divided by them.

This table of relative readings was imported into the R software, version 4.4.3 (R Development Core Team, 2011; http://cran.r-project.org, accessed on 12 January 2026) for further statistical analysis using the phyloseq R package [27]. Differences in diversity indices α were assessed using Student’s *t*-test for pairwise comparison (FM vs. C). The value of *p* < 0.05 was considered significant. Finally, the differential abundance of ZOTU between the control and FM samples was evaluated using the DESeq2 R package [28], considering the adjusted *p*-value (FDR) < 0.05 significant.

### 2.11. Statistical Analysis

All data have been expressed as the mean value of the sample ± standard deviation (SD). In all cases, during the hypothesis testing between groups, the absence of differences between the compared groups has been considered null hypothesis (H_0_), establishing the statistical significance to be able to reject the hypothesis at 0.05 (type I error, α = 0.05). Therefore, whenever the statistical tests applied the *p*-value was lower than the type I error (*p* < 0.05), H_0_ was rejected, i.e., it was assumed that there were statistically significant differences between the groups compared.

To determine the distribution of the data, they were analyzed using the Kolmogorov-Smirnov test before each comparative analysis, thus checking whether the data followed a normal distribution. If so, and if the comparison to be made was of one group versus another, the difference between the means of the compared groups was made using Student’s parametric *t*-test for unpaired measures. On the other hand, if the data did not follow a normal distribution, these differences were assessed with the non-parametric Mann-Whitney U test. The comparison between multiple groups was made with the statistical tests ANOVA of one factor (if the groups followed a normal distribution) or Kruskal Wallis (otherwise). If the differences were statistically significant, the Tukey, Dunn or Dunnett post-hoc tests were used, depending on the comparisons made.

On the other hand, when the correlation between variables was to be assessed, the Pearson test (if the data came from a normal distribution) or the Spearman test (if the data came from a different distribution than normal) was applied. In the case of the comparison of nominal categorical variables, the statistical test chosen to test hypotheses was Fisher’s exact test or Chi-square test.

## 3. Results

### 3.1. Sample and Characteristics of the Subjects

Of the 250 subjects included, 190 completed the study (Figure 1). The sample was composed of women with a mean age of 51.7 years. Additional body measurements (weight, height and BMI) were recorded in Supplementary Table S1. A preliminary analysis of patient characteristics prior to intervention showed no differences between study groups in antibiotics/antioxidants consumption, complex chronic illnesses (autoimmune, cancer, cardiovascular or metabolic) or chronic fatigue (Table 1). However, some other variables less linked to FM and intestinal microbiota did show slight differences.

Furthermore, no differences were observed across timepoints in any of the recorded clinical data (SF-36, FIQR), in either the control group or FM patients.

### 3.2. Microbial Diversity and Richness

**Figure 3:**
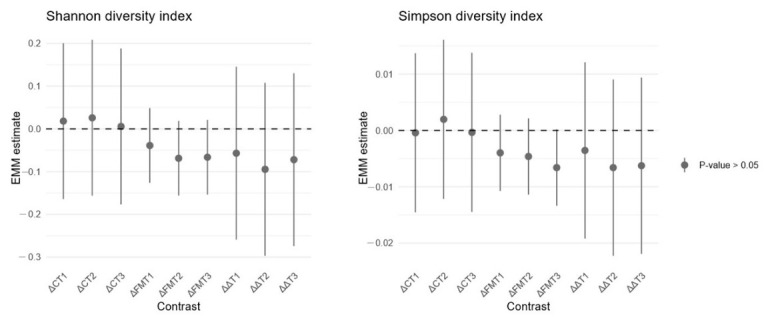
Effect of treatment vs. placebo on Shannon and Simpson diversity indexes of control subjects and FM patients at each time point. Relative differences were calculated within conditions (ΔC or ΔFM) and timepoints (1, 2 and 3 against basal timepoint 0), as well as between conditions (ΔΔ, FM-C) at each time point.

Studying the Shannon and Simpson diversity index at baseline, a statistically significant difference was observed between FM patients and volunteers in T0, as previously published [29]. However, no statistically significant differences in diversity and richness were detected between the groups over time, after applying the different treatments (Figure 3).

### 3.3. Metagenomics Study

**Figure 4:**
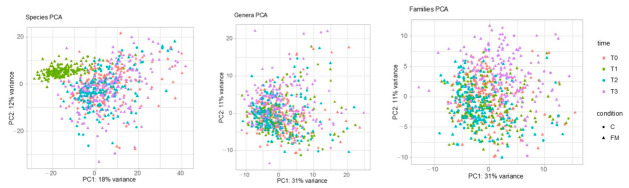
Principal Component Analysis (PCA): evolution of groups over time. From left to right: analysis by species, genera, and families.

A Principal Component Analysis (PCA) for species, genera, and families was performed to analyze data distribution (Figure 4). It was observed that in T1 there were well-defined characteristics that indicate a greater similarity of species for the FM group, while for T0, T2 and T3 the data resulted more dispersed for the two treatment groups, showing a greater interindividual variability of the data. As for the bacterial genera and families, PCA showed an unclear pattern.

**Figure 5:**
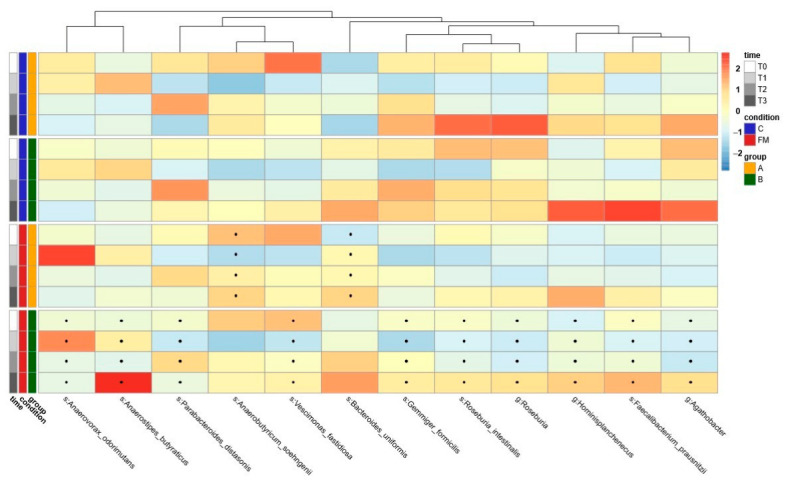
Differences in microbial group abundance depending on time (T0, T1, T2, T3), condition (FM vs. C), and experimental group (A vs. B). Black points indicate adjusted *p*-value < 0.05 when compared with Control samples in each specific time point and treatment/placebo group. Only bacterial species that were consistently detected across all time points and controls, and showed statistically significant differences in at least one comparison, were included in the analysis.

As shown in the heatmap (Figure 5), FM patients who received a placebo (group A) showed a punctual and significant reduction in *Anaerobutyricum soehngenii* at T1. In this group, *Bacteroides uniformis* increased progressively throughout the study. In treated patients (group B), *Anaerovorax odorimutans* showed a punctual and significant increment in T1. Other taxa, such as *Anaerostipes butyraticus* and *Parabacteroides distasonis*, show irregular behavior in these patients across timepoints. In the case of the genus *Hominisplanchenecus*, its abundance increased and remained constant during treatment, reaching its peak value in T3.

Analyzing the abundance for the rest of the taxa in treated patients, a similar pattern was found, being lower during the treatment (T1 and T2) when compared with baseline (T0) and the end of the study (T3) (Figure 5).

**Figure 6:**
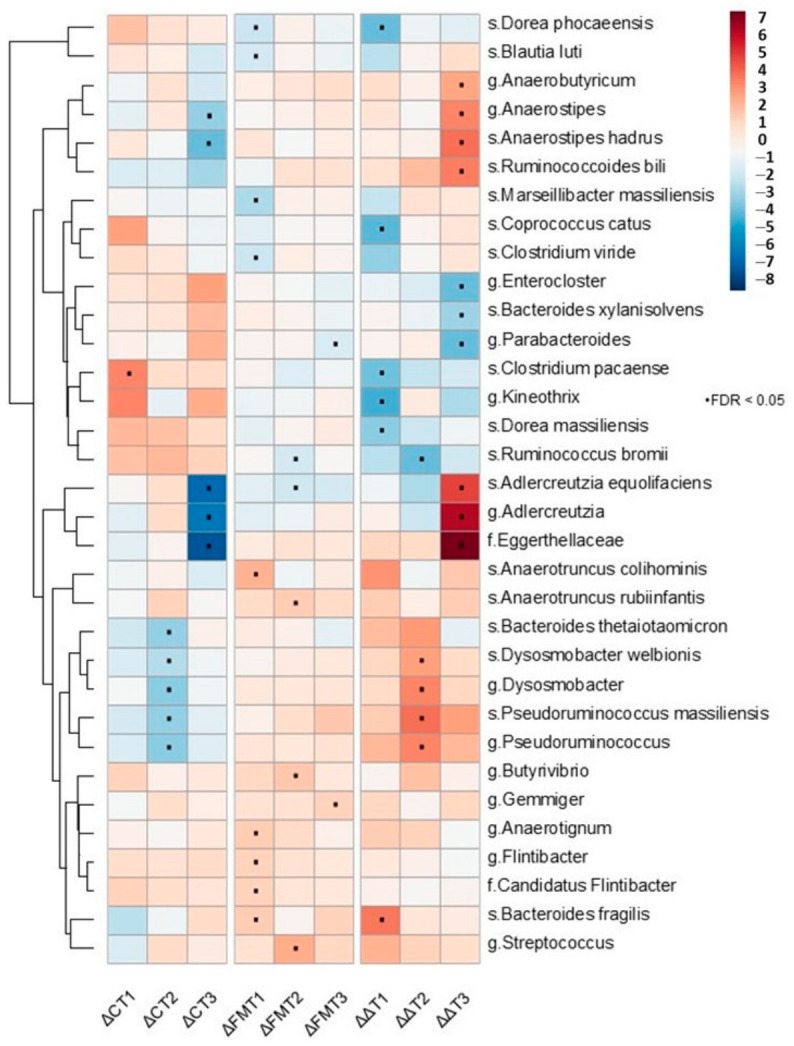
Differences in relative abundance of bacterial species of the gut microbiota within the same group (Δ) compared with the baseline and comparing FM vs. C (ΔΔ). Each column shows the difference in relative expression between two specific conditions, organized by group (fibromyalgia patients [FM] and controls [C]) and time (T0, T1, T2, T3). Black points indicate adjusted *p*-value < 0.05 when compared to T0 within the same group (Δ) or when FM is compared with C subjects (ΔΔ). Only bacterial species consistently detected across all time points and controls were included in the analysis.

Finally, an analysis of differences in treatment vs. placebo in each time point for each group, taking the baseline as reference, was performed (Figure 6). In control subjects, after treatment with EVOO, a statistically significant increase in *Clostridium pacaense* at T1 (3 months of intervention) was found. At T2 (6 months of intervention), *Bacteroides thetaiotaomicron*, *Dysosmobacter* (genus and species), and *Pseudoruminococcus* (genus and species) showed a significant decrease. However, the most significant change in this group is observed in T3, with a pronounced decrease in *Adlercreutzia* (genus and species) and Eggerthellaceae.

Treated FM patients (group B) had higher abundances for *Anaerotruncus colihominis*, *Anaerotignum*, *Flintibacter*, *Candidatus Flintibacter*, and *Bacteroides fragilis*, and lower abundances in *Dorea phocaeensis*, *Blautia luti*, *Marseillibacter massiliensis*, and *Clostridium viride* in T1 (3 months of intervention). In T2, a significant decrease in *Ruminococcus equolifaciens* and *Adlercreutzia equolifaciens* was observed in those patients, while *Anaerotruncus rubiinfantis*, *Butyrivibrio*, and *Streptococcus* were increased.

When comparing the differential effect of EVOO treatment between controls and FM patients (ΔΔ), greater decreases in *Dorea phocaeensis*, *Blautia luti*, *Marseillibacter massiliensis*, and *Clostridium viride* were found in FM patients when compared with control subjects at T1. The opposite trend was found in *Anaerotruncus colihominis*, *Anaerotignum*, *Flintibacter*, *Candidatus Flintibacter*, and *Bacteroides fragilis*, which showed higher levels in patients.

At T2, a more pronounced decrease in *Ruminococcus bromii* and a greater increase in *Dysosmobacter* (both genus and species) and *Pseudoruminococcus* (both genus and species) were observed in FM patients than in volunteers. By the end of the study (T3), *Enterocloster*, *Bacteroides xylanisolvens*, and *Parabacteroides* had lower abundances in patients, while *Anaerobutyricum*, *Anaerostipes*, *Anaerostipes hadrus*, and, especially in *Ruminococcoides bili*, *Adlercreutzia equolifaciens* and Eggerthellaceae family were increased. As shown in Figure 6, this pattern is explained by a marked reduction in these bacterial taxa in the control group following the end of the nutritional intervention, whereas in patients, their abundance remained relatively stable, with only slight changes.

## 4. Discussion

This study shows that a MD enriched with EVOO can modulate key gut bacteria in women with fibromyalgia. Although overall diversity remained stable within each study group, several beneficial taxa increased during the intervention, suggesting a targeted microbiome shift that could support symptom relief through anti-inflammatory and metabolic pathways. However, these results should be interpreted considering that baseline heterogeneity between groups in some clinical and pharmacological variables may have influenced microbiota profiles.

No significant differences were observed across timepoints in any of the recorded clinical outcomes (SF-36, FIQR), despite the presence of relevant differences in the intestinal microbiota profile between groups. This lack of clinical change may be related to the relatively modest microbiome shifts observed and the limited duration of the intervention, which may not be sufficient to induce measurable changes in patient-reported outcomes. In addition, these questionnaires may not be sensitive enough to detect subtle clinical improvements associated with microbiota modulation. Therefore, although microbiota changes were observed, their translation into clinically perceptible effects may require longer follow-up periods or larger effect sizes. The exploratory nature of the study may also partly explain the lack of concordance between microbiological and clinical findings.

Individuals from control group had greater diversity and richness of microbial species than patients with FM. Although some authors found no significant differences in either alpha or beta diversity of FM patients versus a control group [30], these differences have been demonstrated in several FM cohorts. For instance, Kim et al. found lower diversity in FM patients [31] and, similarly, a lower bacterial diversity has been demonstrated in patients with FM, specifically in the genera *Bifidobacterium* and *Eubacterium* [32]. Recent data indicate that FM could be linked to alterations in the gut microbiota and mitochondrial metabolism, which would suggest that it has a crucial role of the gut-mitochondrial axis in the pathophysiology of the disease [33]. Within these microbial changes, a decrease in bacterial diversity was observed, along with an imbalance in the Firmicutes/Bacteroidetes ratio, a common feature in various inflammatory and metabolic conditions [34]. Even though most data suggest the presence of a lower bacterial diversity in FM, additional studies are needed to be able to elucidate this point.

FM patients treated with EVOO experienced a significant increment of *Bacteroides fragilis*, especially at 3 months of intervention. In this sense, it is known that some species of the genus *Bacteroides* have a highly conserved glutamate decarboxylase (GAD) system, which allows them to produce γ-aminobutyric acid (GABA) from glutamate and glutamine. GABA serves as a protective response to acid stress in both bacterial and host cells [35], providing beneficial effects. Within the genus *Bacteroides, Bacteroides fragilis* stands out as one of the most active species in this process [36]. In patients with FM, an alteration in the abundance of *Bacteroides* has been reported, which could be associated with a disruption in GABA production that would contribute to the symptoms of chronic pain and sleep disturbances characteristic of the disease. In line with these results, other studies have detected lower abundances of *Bacteroides uniformis* in this population [9]. In our study, the placebo-treated FM group experienced a progressive increase in *Bacteroides uniformis* throughout the study, an effect that might serve as supporting evidence to reinforce the beneficial role of the MD in intestinal microbiota, apart from the olive oil used as supplement.

Among the bacteria that produce short-chain fatty acids, our results showed that species such as *Faecalibacterium prausnitzii* or *Roseburia intestinalis* were lowered by EVOO treatment in FM patients when compared with placebo, with a rebound effect at the end of the study (T3, 6 months after nutritional intervention). A study has identified several OTUs in people with FM with a different abundance than those found in healthy subjects. Among them, some beneficial taxa such as *Faecalibacterium prausnitzii* or *Bacteroides uniformis* appeared decreased in patients with FM [9]. Since the higher abundance was found in the FM group treated with EVOO, this supplementation could have a protective role in the intestinal microbiota of FM patients, but only effective in the post-treatment phase, weeks after the intervention. As Nie et al. point out in their review [37], dysbiosis of *Roseburia intestinalis,* a butyrate-producing bacterium, can negatively affect different body systems, favoring the development or maintenance of some diseases such as cystic fibrosis, Parkinson’s, depression, inflammatory bowel disease, diabetes, infectious diseases, antiphospholipid syndrome or atherosclerosis. In vitro and animal studies have shown improvements in inflammation following the administration of *R. intestinalis,* suggesting that it could be an effective treatment for patients with inflammatory bowel disease [38]. Also in animals, it has been observed that the butyrate that produces *R. intestinalis* could relieve pain by modulating the vagus nerve, thus playing a key role in the gut-brain axis [39].

Notably, in a pilot study by our team, it was observed that there was a higher abundance of *Ruminococcus* spp. in female patients when compared with male patients, showing both a marked mitochondrial imbalance and a differential intestinal microbiota [33]. This abnormal mitochondrial disturbance was found previously in a different cohort of FM patients [40], suggesting an alteration in cellular energy production. The role of mitochondrial-microbiota axis has been depicted in several areas, such as aging, neurodegeneration and exercise [41,42,43]. Since gut microbiota is essential in the regulation of energy metabolism, the dysbiosis observed in FM could be affecting mitochondrial homeostasis, contributing to the chronic fatigue and muscle dysfunction reported in these patients [33]. However, the present study does not allow determination of whether these changes are a cause or a consequence of the clinical symptoms.

In the results of this study, in more than 200 women, the abundance of *Ruminococcus bromii* was reduced in FM patients supplemented with EVOO, when compared to volunteers, at 6 months of treatment. In contrast, in this group, *Ruminococcus bili* shows a progressive increase throughout the study. Among the diffuse symptoms that people with FM may suffer from are some digestive disorders. In this sense, a study carried out in individuals with inflammatory bowel disease highlighted the possible beneficial role of some species of *Ruminococcus* for their intestinal health [44]. The findings observed point to a possible relationship between increased *Ruminococcus* and an improvement in digestive symptoms in certain FM patients, although further research is needed to establish this association more robustly.

On the other hand, the results of the present study have shown that the *Streptococcus* genus experiences a significant increase after 6 months of intervention in FM patients treated with EVOO. However, in previous studies, the low abundance of some species belonging to this genus has been associated with beneficial effects on host health. For example, *S. salivarius* or *S. mutans* appear decreased in people with good oral health [45]. Strikingly, higher abundances of *Streptococcus* bacteria have been related to gastrointestinal issues, such as functional dyspepsia [46]. For this reason, the reasons and the consequences of the rise of these taxa in FM patients that were treated with EVOO in this study need to be addressed, since it could be taken as a negative effect.

During the study intervention, the *Agathobacter* genus remained at low levels in FM patients, while at 12 months (6 months after the end of the intervention) its levels increased significantly. Overall, *Agathobacter* appears to have a beneficial effect on gut health, as it can produce short-chain fatty acids such as butyrate, propionate, and lactate [47,48]. In fact, in malnourished children who were supplemented with a food that targeted microbiota, an increase in the fecal abundance of *Agathobacter* was observed [48]. In another study, supplementing with a seasoning similar to soy sauce increased the abundance of *Agathobacter rectalis* and, with it, the production of short-chain fatty acids [47]. Similarly, the fecal microbiota of patients with inflammatory bowel disease with extraintestinal symptoms lacks bacteria from the Lachnospiraceae family, such as *Agathobacter* and *Blautia* [49]. In a cross-sectional study conducted in women with sarcopenia, it was observed that the presence of *Agathobacter* was positively correlated with grip strength [50]. Likewise, *Agathobacter* also appears in greater abundance in healthy people compared to patients with gout [51]. These results could indicate that the intervention carried out in the present study requires a few months to exert the expected beneficial effect and modify the intestinal microbiota significantly.

Another bacterial genus that produces butyrate is *Anaerostipes*. The results of our study show that *Anaerostipes butyraticus* has an erratic behavior throughout the study in FM patients treated with EVOO. However, when these results are compared with those obtained for control subjects, it is observed that, in T3, there are greater increases in the relative abundance of the genus *Anaerostipes* and, specifically, of *Anaerostipes hadrus* in FM, while those levels suffer a dramatic fall in control subjects 6 months after the intervention. The abundance of this genus could be relevant for the control of insulin resistance, due to its butyrate production. In fact, it has been observed that patients with type 2 diabetes have less of these bacteria than healthy people [52].

The abundance of *Parabacteroides distasonis* also shows variable behavior in FM people treated with EVOO. This species has been studied in different pathologies and may be a positive factor for the prevention or treatment of some of them. In mice with inflammatory arthritis, oral treatment with *P. distasonis* in the form of a probiotic significantly improved the pathogenesis of the disease [53]. In mice with non-alcoholic steatohepatitis, treatment with inulin protected the animals from the disease, and it seems that *P. distasonis* was responsible for using this substance to produce pentadenoic acid, a fatty acid that favors the reduction of hepatic steatosis, necroinflammation, abdominal distension and fibrosis [54]. In humans, it has been observed that the presence of *P. distasonis* is inversely correlated with insulin resistance, becoming a possible tool to prevent and treat this condition [53]. However, a 2021 review shows that the role of *P. distasonis* is controversial. In some studies, it appears to have a beneficial role for host health (colorectal cancer or obesity), while in others it appears to harm host health (inflammatory bowel disease, diabetes, autoimmune diseases, or cardiovascular disease) [55]. Although *P. distasonis* could be considered as an important candidate for the development of new probiotics, it should not be forgotten that this genus has shown resistance to some antibiotics (clindamycin, moxifloxacin and cefoxitin) [56]. All this suggests that *P. distasonis* could play a relevant role in the health of individuals, although it would be necessary to develop studies that justify their role in the treatment of FM.

At the end of the study, 6 months after the end of the intervention (T3), a more significant increase in the abundance of the family Eggerthellaceae, and specifically of the genus *Adlercreutzia* and the species *Adlercreutzia equolifaciens,* was observed in FM patients treated with EVOO than in controls. *A. equolifaciens* has been identified as a key microorganism in the production of equol, a metabolite with potent antioxidant and estrogenic effects [57]. In a recent study, this species was significantly more abundant in people able to produce equol, who in turn had a lower prevalence of dyslipidemia and lower triglyceride levels, suggesting a beneficial role in the regulation of lipid metabolism [58]. These results support the possible involvement of this bacterium and equol in the modulation of systemic inflammatory states and metabolism, which could have relevance in the context of FM, a disease with inflammatory and metabolic components. Likewise, this bacterium has been shown to modulate the NF-κB inflammatory pathway in in vitro and in vivo models, in addition to reducing the expression of interleukin-6 in intestinal and liver tissue in mice, as well as generating beneficial metabolic changes such as a decrease in body weight, transient reduction of hyperglycemia and increase in butyrate, thus associating its presence in the intestinal microbiome with a eubiotic state [59]. In humans, the abundance of this species is significantly reduced in patients with non-alcoholic hepatic steatosis (NAFLD) [59]. All this suggests that this species could have a protective role against chronic diseases with an inflammatory basis, among which could be FM.

The most relevant changes associated with EVOO consumption in FM patients are summarized in Table 2.

**Table 2:** Summary of the significant differential changes observed with EVOO treatment in FM patients.

Taxon	Observed Change	Link with FM	References
*Bacteroides fragilis*	↑ ΔΔT1 (differential increment with EVOO in FM patients when compared with EVOO in C)	GABA producer; involved in gut–brain axis modulation and chronic pain relief	[35,36]
*Anaerotruncus colihominis*	**↑** ΔFMT1 (differential increment with EVOO in FM patients when compared with FM baseline)	Associated with metabolic regulation; increased abundance linked to EVOO treatment	[60]
*Ruminococcus bromii*	**↓** ΔΔT2 (differential reduction with EVOO in FM patients when compared with EVOO in C)	Decrease in FM may relate to altered fiber degradation; contextual role not clear	[33]
*Pseudoruminococcus*	**↑** ΔΔT2 (differential increment with EVOO in FM patients when compared with EVOO in C)	Linked to fiber metabolism and short-chain fatty acid (SCFA) production	[61]
*Dysosmobacter*	**↑** ΔΔT2 (differential increment with EVOO in FM patients when compared with EVOO in C)	Emerging genus associated with lipid metabolism modulation	[62]
*Streptococcus*	**↑** ΔFMT2 (differential increment with EVOO in FM patients when compared with FM baseline)	Mixed effects; some species linked to immune modulation and inflammation	[45,63]
*Anaerostipes hadrus*	**↑** ΔΔT2 (differential increment with EVOO in FM patients when compared with EVOO in C)	Butyrate producer; potential benefit for insulin resistance	[52]

## 5. Conclusions

The results suggest that dietary intervention based on the MD supplemented with EVOO could partially modulate the composition of the gut microbiota in women with FM. Although no significant changes were observed in the overall indices of microbial diversity after the intervention within each study group, specific and subtle modifications were detected in the relative abundance of several bacterial taxa associated with relevant metabolic functions, such as the production of short-chain fatty acids and the modulation of the immune system.

After 3 months of EVOO treatment, an increase in species such as *Bacteroides fragilis*, *Anaerotruncus colihominis* and *Adlercreutzia equolifaciens*, whose metabolic activity could be involved in antioxidant and anti-inflammatory mechanisms, was observed in FM patients. Furthermore, the pattern observed for *Faecalibacterium prausnitzii*, *Roseburia intestinalis* and *Agathobacter* might suggest a possible delayed or sustained effect of EVOO treatment on intestinal microbial composition.

These findings must be classified as preliminary but reinforce the hypothesis that specific nutritional interventions may have a relevant impact on the gut microbiota of FM patients.

## 6. Limitations and Future Perspectives

This study has several limitations that should be considered. First, the cohort is characterized by a high prevalence of polypharmacy, which may act as a potential confounding factor influencing gut microbiota composition and limiting the attribution of observed changes exclusively to dietary intervention. In addition, although dietary recommendations were personalized, adherence to a semi-structured MD may have varied between participants, introducing additional variability in the results. The last limitation about the cohort is the relatively homogeneous sample, composed exclusively of women with fibromyalgia, limits the generalizability of the findings to other populations, including men or patients with different disease severities.

From a methodological perspective, the use of 16S rRNA gene sequencing represents a technical limitation, as it restricts taxonomic resolution and does not allow direct assessment of microbial functionality. Therefore, the functional implications of the observed taxonomic shifts cannot be fully determined.

Future studies should aim to control pharmacological variables more strictly, improve dietary adherence monitoring, and incorporate multi-omics approaches to better characterize the functional impact of microbiota modulation in fibromyalgia.

## Figures and Tables

**Table 1:** Characteristics of patients before the intervention.

Covariate	*p*-Value	*p*-Value adj	Q-Value	Significance	C	FM	Test
Alcohol	8.00 × 10^−5^	3.70 × 10^−4^	1.90 × 10^−4^	yes	76.74%	42.21%	Chi-square
Analgesics	5.40 × 10^−13^	1.20 × 10^−11^	6.40 × 10^−12^	yes	13.95%	73.87%	Chi-square
Antibiotics	1.00	1.00	0.51	no	0.00%	0.50%	Fisher’s exact
Antidepressants	1.10 × 10^−10^	1.30 × 10^−9^	6.60 × 10^−10^	yes	4.65%	60.30%	Chi-square
Antioxidants	0.08	0.13	0.07	no	0.00%	8.04%	Fisher’s exact
Anxiety depressive	8.00 × 10^−5^	3.70 × 10^−4^	1.90 × 10^−4^	yes	65.12%	89.95%	Chi-square
Autoimmune	0.31	0.40	0.20	no	6.98%	14.07%	Chi-square
Cancer	0.80	0.83	0.43	no	4.65%	2.51%	Chi-square
Cardiovascular	0.08	0.13	0.07	no	4.65%	16.58%	Chi-square
Chronic fatigue	0.22	0.30	0.15	no	0.00%	5.53%	Fisher’s exact
Depression	2.80 × 10^−8^	2.10 × 10^−7^	1.10 × 10^−7^	yes	2.33%	49.75%	Chi-square
Dermatologic	0.07	0.13	0.06	no	4.65%	17.09%	Chi-square
Digestive	1.91 × 10^−3^	0.01	3.21 × 10^−3^	yes	9.30%	34.67%	Chi-square
Digestive symptoms	0.01	0.02	0.01	yes	93.02%	100.00%	Fisher’s exact
Endometriosis	0.09	0.13	0.07	no	4.65%	16.08%	Chi-square
Gynecologic	0.04	0.08	0.04	yes	9.30%	25.63%	Fisher’s exact
Inflammatory	0.04	0.08	0.04	yes	2.33%	14.57%	Fisher’s exact
Metabolic	0.15	0.21	0.11	no	4.65%	14.07%	Chi-square
Musculoskeletal	6.30 × 10^−4^	2.41 × 10^−3^	1.23 × 10^−3^	yes	6.98%	34.67%	Chi-square
Neurological	0.01	0.02	0.01	yes	2.33%	20.10%	Chi-square
Sedentarism	0.45	0.54	0.28	no	46.51%	54.27%	Chi-square
Smoker	0.56	0.62	0.32	no	11.63%	16.58%	Chi-square
Specific diet	0.56	0.62	0.32	no	20.93%	29.15%	Fisher’s exact

## Data Availability

The datasets are publicly available. The metagenomic data are uploaded in GSA-Human under the accession number HRA009721 (BioProject accession: PRJCA032811), available at https://bigd.big.ac.cn/gsa-human/browse/HRA009721, accessed on 12 January 2026. The proteomic data are deposited in the PRIDE database under the ProteomeXchange accession number PXD059894, with the project webpage available at https://www.ebi.ac.uk/pride/archive/projects/PXD059894, accessed on 12 January 2026 and the FTP download at https://ftp.pride.ebi.ac.uk/pride/data/archive/2025/09/PXD059894, accessed on 12 January 2026.

## Supplementary Materials

The following supporting information can be downloaded at: https://www.mdpi.com/article/10.3390/life16060894/s1, File S1: Clinical questionnaires used in FM (SF-36 and FIQR); Table S1. Additional body measurements (weight, height and BMI).
