# Supplementary material for: Impact of a Mediterranean Diet Supplemented with Extra Virgin Olive Oil on Gut Microbiota in Fibromyalgia: A Randomized Controlled Trial
Source: Life (Basel). 2026 May 26;16(6):894. doi: 10.3390/life16060894 (PMC13301056; doi:10.3390/life16060894)
Supplement: Supplementary file 1 [file life-16-00894-s001.zip › supplementary.table.S1.xlsx - supplementary.table.S1.pdf]

Supplementary Table S1: Additional body measurements (weight, height and BMI).

Code	Weight (Kg)	Height (cm)	IMC	Group	Condition
1	51,00	157,00	20,69	B	FM
2	53,00	160,00	20,70	A	FM
3	54,00	165,00	19,83	B	FM
4	54,00	157,00	21,91	B	FM
5	55,00	166,00	19,96	B	FM
6	56,00	155,00	23,31	A	FM
7	57,00	164,00	21,19	B	FM
8	58,00	159,00	22,94	A	FM
9	59,00	149,00	26,58	B	FM
14	64,00	157,00	25,96	A	FM
15	64,00	160,00	25,00	A	FM
16	64,00	174,00	21,14	A	FM
17	65,00	165,00	23,88	A	FM
18	68,00	157,00	27,59	B	FM
19	68,00	165,00	24,98	A	FM
20	69,00	160,00	26,95	A	FM
21	69,00	170,00	23,88	B	FM
23	70,00	161,00	27,01	A	FM
24	70,00	165,00	25,71	B	FM
25	73,00	178,00	23,04	B	FM
26	75,00	164,00	27,89	A	C
27	77,00	158,00	30,84	A	FM
28	77,00	155,00	32,05	A	FM
29	80,00	163,00	30,11	A	FM
30	83,00	160,00	32,42	B	FM
31	88,00	165,00	32,32	A	FM
32	95,00	168,00	33,66	A	FM
34	73,00	170,00	25,26	A	FM
35	85,00	163,00	31,99	B	FM
36	74,00	152,00	32,03	B	FM
37	75,00	163,00	28,23	A	FM
39	49,50	161,50	18,98	A	FM
40	57,00	169,00	19,96	B	FM
41	53,00	158,00	21,23	B	FM
42	56,00	160,00	21,88	A	FM
43	80,00	165,00	29,38	A	FM
44	64,00	169,00	22,41	B	FM
46	60,00	161,00	23,15	A	FM
47	59,00	161,00	22,76	A	FM
49	60,00	161,00	23,15	B	FM
50	60,00	160,00	23,44	A	C
51	60,00	160,00	23,44	A	FM
52	61,00	160,00	23,83	B	FM
53	74,00	176,00	23,89	B	FM

54	65,00	164,00	24,17	A	FM
55	59,00	155,00	24,56	A	FM
57	72,00	170,00	24,91	B	FM
58	73,00	171,00	24,96	A	FM
59	64,00	160,00	25,00	A	FM
60	74,00	170,00	25,61	B	FM
61	70,00	163,00	26,35	B	FM
62	64,00	155,00	26,64	A	FM
63	72,00	164,00	26,77	A	FM
64	76,00	168,00	26,93	B	FM
65	79,00	170,00	27,34	B	FM
66	64,00	153,00	27,34	A	FM
67	73,00	162,00	27,82	A	FM
68	67,00	155,00	27,89	B	FM
69	78,00	167,00	27,97	B	FM
70	76,00	163,00	28,60	A	FM
71	85,00	172,00	28,73	A	FM
72	58,00	161,00	22,38	B	FM
73	68,00	152,00	29,43	B	FM
74	80,00	164,00	29,74	A	FM
75	76,00	157,00	30,83	A	FM
76	75,00	155,00	31,22	B	FM
77	85,00	165,00	31,22	B	FM
78	79,00	159,00	31,25	A	FM
79	79,00	158,00	31,65	A	FM
80	81,00	157,00	32,86	B	FM
81	95,00	170,00	32,87	B	FM
82	65,00	140,00	33,16	A	FM
83	87,00	160,00	33,98	A	FM
84	85,00	157,00	34,48	B	FM
85	76,00	160,00	29,69	B	FM
86	63,00	173,00	21,05	A	FM
87	85,00	164,00	31,60	A	FM
88	90,00	167,00	32,27	B	FM
89	79,00	169,00	27,66	B	FM
90	87,00	165,00	31,96	A	C
91	70,00	158,00	28,04	A	C
92	65,00	155,00	27,06	B	C
94	52,00	167,00	18,65	A	FM
95	53,00	168,00	18,78	A	FM
96	53,00	167,00	19,00	B	FM
97	51,00	162,00	19,43	B	FM
98	57,00	168,00	20,20	A	FM
99	57,00	165,00	20,94	A	FM
100	63,00	173,00	21,05	B	FM
101	57,00	164,00	21,19	B	FM
102	58,00	164,00	21,56	A	FM

103	60,00	165,00	22,04	A	FM
104	60,00	164,00	22,31	B	FM
105	60,00	163,00	22,58	B	FM
106	61,00	163,00	22,96	A	FM
107	57,00	157,00	23,12	A	FM
108	56,00	155,00	23,31	B	FM
109	60,00	160,00	23,44	B	FM
110	67,00	167,00	24,02	A	C
111	60,00	158,00	24,03	A	C
113	66,00	162,00	25,15	B	C
115	69,00	160,00	26,95	A	FM
116	72,00	163,00	27,10	B	FM
117	78,00	168,00	27,64	B	FM
118	80,00	170,00	27,68	A	FM
119	72,00	160,00	28,13	A	FM
120	77,00	165,00	28,28	B	C
121	72,00	159,00	28,48	B	C
122	70,00	155,00	29,14	A	FM
123	78,00	163,00	29,36	A	FM
124	75,00	158,00	30,04	B	FM
125	80,00	163,00	30,11	B	FM
126	78,00	160,00	30,47	A	FM
127	85,00	167,00	30,48	A	FM
128	87,00	167,00	31,20	B	FM
130	86,00	165,00	31,59	A	FM
131	89,00	165,00	32,69	A	FM
133	55,00	164,00	20,45	B	FM
135	49,00	160,00	19,14	A	FM
136	62,00	173,00	20,72	B	FM
138	60,00	169,00	21,01	A	FM
139	53,00	158,00	21,23	A	FM
141	57,00	160,00	22,27	B	FM
142	63,00	167,00	22,59	A	FM
143	63,00	165,00	23,14	A	FM
145	64,00	164,00	23,80	B	FM
146	59,00	157,00	23,94	A	FM
147	60,00	158,00	24,03	A	C
148	69,00	168,00	24,45	B	FM
149	69,00	168,00	24,45	B	C
150	59,00	155,00	24,56	A	FM
152	73,00	170,00	25,26	B	FM
153	75,00	169,00	26,26	B	FM
154	72,00	163,00	27,10	A	FM
155	79,00	169,00	27,66	A	FM
157	68,00	153,00	29,05	B	FM
158	72,00	157,00	29,21	A	C

159	80,00	165,00	29,38	A	FM
160	71,00	153,00	30,33	B	FM
161	72,00	154,00	30,36	B	FM
162	76,00	156,00	31,23	A	FM
163	85,00	160,00	33,20	A	FM
164	85,00	160,00	33,20	B	FM
165	95,00	168,00	33,66	B	FM
166	85,00	165,00	31,22	A	FM
167	68,00	159,00	26,90	A	FM
168	51,00	162,00	19,43	B	FM
169	80,00	172,00	27,04	B	FM
170	64,00	163,00	24,09	A	FM
171	83,00	164,00	30,86	A	FM
172	55,00	160,00	21,48	B	FM
174	64,00	160,00	25,00	A	FM
175	68,00	170,00	23,53	A	FM
176	57,00	162,00	21,72	B	FM
177	88,00	170,00	30,45	B	FM
178	48,00	156,00	19,72	A	FM
179	90,00	170,00	31,14	A	FM
180	51,00	158,00	20,43	B	FM
181	55,00	163,00	20,70	B	FM
182	62,00	173,00	20,72	A	C
183	46,00	147,00	21,29	A	FM
185	57,00	158,00	22,83	A	FM
186	64,00	167,00	22,95	A	FM
188	59,00	160,00	23,05	B	FM
189	62,00	162,00	23,62	B	FM
190	60,00	159,00	23,73	A	FM
192	56,00	153,00	23,92	A	FM
193	61,00	159,00	24,13	B	FM
194	65,00	164,00	24,17	A	FM
195	65,00	164,00	24,17	A	FM
196	70,00	170,00	24,22	B	FM
197	70,00	168,00	24,80	B	FM
198	64,00	160,00	25,00	A	FM
199	72,00	169,00	25,21	A	C
200	67,00	163,00	25,22	B	FM
201	65,00	159,00	25,71	B	C
202	75,00	170,00	25,95	A	FM
203	67,00	158,00	26,84	A	FM
205	70,00	158,00	28,04	B	FM
206	77,00	165,00	28,28	A	FM
208	74,00	161,00	28,55	B	FM
209	77,00	164,00	28,63	B	FM
210	78,00	165,00	28,65	A	FM

211	68,00	152,00	29,43	A	FM
212	81,00	164,00	30,12	B	C
215	78,00	160,00	30,47	A	FM
216	82,00	164,00	30,49	B	FM
219	155,00	78,00	32,47	B	FM
221	150,00	74,00	32,89	B	FM
222	105,00	174,00	34,68	B	FM
223	57,00	153,00	24,35	A	FM
225	78,00	167,00	27,97	A	FM
227	53,00	167,00	19,00	B	C
229	60,00	173,00	20,05	B	C
230	53,00	162,00	20,20	B	FM
231	55,00	163,00	20,70	A	FM
232	50,00	153,00	21,36	B	FM
236	64,00	169,00	22,41	A	FM
237	54,00	155,00	22,48	B	C
228	58,00	171,00	19,84	A	C
239	57,00	157,00	23,12	A	C
240	68,00	170,00	23,53	B	FM
241	64,00	164,00	23,80	B	FM
242	60,00	158,00	24,03	B	FM
243	73,00	172,00	24,68	B	FM
244	70,00	168,00	24,80	A	C
250	73,00	165,00	26,81	A	FM
251	75,00	167,00	26,89	A	FM
252	76,00	167,00	27,25	B	FM
253	75,00	165,00	27,55	A	FM
254	72,00	160,00	28,13	A	FM
255	75,00	163,00	28,23	A	FM
256	90,00	175,00	29,39	A	FM
257	76,00	160,00	29,69	B	C
259	93,00	175,00	30,37	B	FM
260	78,00	160,00	30,47	A	FM
261	85,00	166,00	30,85	A	FM
262	85,00	165,00	31,22	B	FM
263	85,00	162,00	32,39	B	FM
264	98,00	172,00	33,13	A	FM
265	92,00	166,00	33,39	B	C
266	82,00	156,00	33,69	B	FM
267	92,00	158,00	36,85	A	C
268	70,00	175,00	22,86	B	FM
269	80,00	160,00	31,25	B	FM
270	81,00	161,00	31,25	B	FM
271	67,00	156,00	27,53	B	FM
273	56,00	168,00	19,84	B	FM
274	52,30	164,00	19,45	B	C

275	60,00	159,00	23,73	B	C
276	59,00	154,00	24,88	A	FM
277	84,00	160,00	32,81	A	FM
278	60,00	170,00	20,76	B	C
279	63,00	163,00	23,71	A	FM
280	63,00	160,00	24,61	A	C
281	58,00	161,00	22,38	A	C
282	75,00	162,00	28,58	A	C
285	72,00	165,00	26,45	B	C
286	58,00	177,00	18,51	A	C
287	50,00	160,00	19,53	B	FM
288	54,00	156,00	22,19	B	C
289	67,00	154,00	28,25	B	C
291	54,00	156,00	22,19	A	C
292	60,00	162,00	22,86	B	C
294	67,00	164,00	24,91	A	C
295	71,00	157,00	28,80	B	C
296	72,00	167,00	25,82	B	C
298	65,00	168,00	23,03	B	C
299	51,00	160,00	19,92	B	C
300	51,00	154,00	21,50	B	C
