# Supplementary material for: Impact of a Mediterranean Diet Supplemented with Extra Virgin Olive Oil on Gut Microbiota in Fibromyalgia: A Randomized Controlled Trial
Source: Life (Basel). 2026 May 26;16(6):894. doi: 10.3390/life16060894 (PMC13301056; doi:10.3390/life16060894)
Supplement: Supplementary file 1 [file life-16-00894-s001.zip › Tiempo 0 - Formularios de Google - Supplementary.material.s1.questionnaires.pdf]

Supplementary Material File S1: Clinical questionnaires used in FM (SF-36 and FIQR).

CUESTIONARIO TIEMPO CERO

Este formulario debe ser completado de forma detallada para su correcto procesamiento. La duración estimada del mismo es de entre 20 y 30 minutos.
En PRONACERA THERAPEUTICS, S.L., como responsables, tratamos la información que usted nos facilita con el fin de realizar un cribado basado en los datos clínicos y demográficos que usted nos facilita a través del mismo. Este cribado nos permitirá recopilar la información necesaria para nuestra línea de Investigación y Desarrollo relacionada con las bases moleculares de la fibromialgia. La base legítima es el consentimiento expreso. La información puede ser cedida a los partners científicos y médicos con los que colaboramos activamente en esta línea, únicamente con fines de investigación.
Usted puede ejercer sus derechos ante PRONACERA THERAPEUTICS, S.L. en C/ MIRO 11, 41804, OLIVARES (SEVILLA), en el correo electrónico info@pronacera.com

* Indica que la pregunta es obligatoria

CUADERNO DE RECOGIDA DE DATOS

1. Nombre *

____

2. Apellidos *

____

3. Número de teléfono *

____

4. Código asignado en el proyecto *

Ejemplo: 0009, 0027

____

5. Fecha de nacimiento *

Formato: __/__/_

6. ¿Es usted mujer? *

Marca solo un óvalo.

- Sí
- No

7. Lugar de nacimiento *

8. Lugar de residencia *

9. ¿Tiene usted diagnóstico clínico de fibromialgia (informe)? *

Marca solo un óvalo.

- Sí
- No

10. Indique el criterio de diagnóstico de fibromialgia:

Marca solo un óvalo.

- ACR 1990
- ACR 2011
- ACR 20106
- No lo sé

11. ¿Algún familiar ha sido diagnosticado de fibromialgia? *

Marca solo un óvalo.

- Sí
- No

12. ¿Está dispuesta a tomar aceite de oliva virgen extra? *

Marca solo un óvalo.

- Sí
- No

13. ¿Está dispuesta a seguir un tratamiento nutricional durante 6 meses con intervención dietética y cambio de hábitos alimentarios? *

Marca solo un óvalo.

- Sí
- No

14. ¿Está dispuesta a realizarse análisis de sangre y de heces en los 4 tiempos designados de estudio (inicio, 3 meses, 6 meses, 12 meses)? *

Marca solo un óvalo.

- Sí
- No

Antropometría

15. Talla (cm) *

Recomendaciones: descalzarse y marcar ligeramente un punto sobre su cabeza en la pared. Luego, use una cinta de medir metálica y mida desde la base del piso hasta la marca en la pared para obtener la medida de la estatura.

16. Peso (kg) *

Recomendaciones: en la mañana, use su báscula de casa, recién levantada, sin ropa, apoye la báscula en una superficie plana y dura (no sobre alfombras o moquetas).

17. Perímetro cintura (cm) *

Recomendaciones: el perímetro abdominal se puede medir fácilmente con una cinta métrica. Así, la persona debe estar de pie, con los pies juntos, los brazos a los lados y el abdomen relajado para, a continuación, rodear su abdomen con la cinta métrica a la altura del ombligo y sin presionar hacer una inspiración profunda y medir al sacar el aire. http://www.portalfitness.com/9028_como-se-mide-el-perimetro-de-la-cintura.aspx

18. En los últimos 3 meses, ¿ha subido/bajado de peso de forma considerable? *

Se entiende como cambio de peso considerable la diferencia de 3-5kg con respecto a los meses previos.

Marca solo un óvalo.

- Sí
- No

Estilo de vida

19. Horas de sueño *

20. Calidad del sueño *

Marca solo un óvalo.

	1	2	3	4	5	6	7	8	9	10	
Muy	◯	◯	◯	◯	◯	◯	◯	◯	◯	◯	Muy buena

21. ¿Tienes estrés? *

Marca solo un óvalo.

◯ Sí

◯ No

22. ¿Tienes ansiedad? *

Marca solo un óvalo.

◯ Sí

◯ No

23. ¿Tienes depresión? *

Marca solo un óvalo.

◯ Sí

◯ No

24. ¿Lleva un estilo de vida sedentario? *

Se entiende como estilo de vida sedentario la falta de actividad física regular, es decir, menos de 30 minutos de ejercicio diario y menos de 3 días a la semana.

Marca solo un óvalo.

- Sí
- No

25. ¿Qué tipo de actividad física realiza? *

____

26. Indique las hora de actividad física semanales *

____

Salud

27. ¿Presenta algún tipo de reacción o sintomatología adversa frente al consumo de aceite de oliva? *

Marca solo un óvalo.

- Sí
- No

28. ¿Tiene otras alergias o intolerancias? *

Marca solo un óvalo.

- Sí
- No

29. Indique las alergias o intolerancias que padece (poner "ninguna" si no es su caso) *

30. ¿Tiene sensibilidad química múltiple? *

Marca solo un óvalo.

- Sí
- No

31. ¿Tiene sensibilidad a electro-magnetismos (electrosmog)? *

Marca solo un óvalo.

- Sí
- No

32. Indique si ha sido diagnosticada de alguna de las siguiente enfermedades *

Selecciona todos los que correspondan.

- [ ] Músculo-esquelética
- [ ] Neurológica
- [ ] Autoinmune (lupus, celiaquía, tiroiditis)
- [ ] Cardiovascular (aterosclerosis, hipertensión, miocardiopatía)
- [ ] Metabólicas (diabetes, síndrome metabólico, hipercolesterolemia)
- [ ] Digestivas (síndrome de intestino irritable, colitis ulcerosa)
- [ ] Inflamatoria (artritis reumatoide, psoriasis)
- [ ] Dermatológicas
- [ ] Ginecológicas
- [ ] Cáncer
- [ ] Ninguna
- [ ] Otras

33. Si son otras las enfermedades, indique cuáles:

____

34. Indique operaciones quirúrgicas relevantes: *

____
____
____
____
____

35. ¿Tiene infecciones repetitivas que requieran la toma de antibióticos? *

Marca solo un óvalo.

- ◯ Sí
- ◯ No

36. Aclare si estas infecciones son respiratorias, de piel, urinarias o ginecológicas:

COVID-19

37. ¿Ha sufrido COVID-19?

Marca solo un óvalo.

- Sí
- No

38. Indique la fecha aproximada de cuando sufrió la COVID-19 o ponga "ninguna" si no es su caso:

39. ¿Está vacunada contra el coronavirus?

Marca solo un óvalo.

- Sí
- No

40. Tipo de vacuna:

Marca solo un óvalo.

- Pfizer
- Moderna
- Astrazeneca
- Combinada
- No estoy vacunada

41. Dosis de la vacuna:

Marca solo un óvalo.

- Una
- Dos
- Tres

Alimentación

42. Si sigue alguna dieta específica indique cuál (poner "ninguna" en caso de no seguirla): *

____

43. ¿Está dispuesta a cambiar su dieta a una dieta mediterránea? *

Marca solo un óvalo.

- Sí
- No

44. ¿Cuántos vasos de agua toma al día? *

____

45. Si toma algún complemento alimenticio indique cuál (poner "ninguno" en caso de no tomarlo): *

____

Medicación

En el caso de tomar medicamentos o estar en tratamiento indíquelo a continuación

46. Antidepresivos

47. Antioxidantes

48. Antinflamatorios

49. Analgésicos

50. Antioxidantes

51. Otros

52. Si suspendió alguna medicación porque no le sienta bien, aclare cuál y la reacción presentada (indique "ninguna" si no es su caso) *

Salta a la pregunta 53

Cuestionario de salud SF-36

INSTRUCCIONES: Conteste cada pregunta tal como se indica y marque una sola respuesta. Si no está seguro/a de cómo responder a una pregunta, por favor conteste lo que le parezca más cierto.

53. 1) En general, usted diría que su salud es: *

Marca solo un óvalo.

- a. Excelente
- b. Muy buena
- c. Buenas
- d. Regular
- e. Mala

54. 2) ¿Cómo diría que es su salud actual, comparada con la de hace un año? *

Marca solo un óvalo.

- a. Mucho mejor ahora que hace un año
- b. Algo mejor ahora que hace un año
- c. Más o menos igual que hace un año
- d. Algo peor ahora que hace un año
- e. Mucho peor ahora que hace un año

Las siguientes preguntas se refieren a actividades o cosas que usted podría hacer en un día normal

55. 3) Su salud actual, ¿le limita para hacer esfuerzos intensos, tales como correr, levantar objetos pesados, o participar en deportes agotadores? *

Marca solo un óvalo.

- a. Sí, me limita mucho
- b. Sí, me limita un poco
- c. No, no me limita nada

56. 4) Su salud actual, ¿le limita para hacer esfuerzos moderados, como mover una mesa, pasar la aspiradora, jugar a los bolos o caminar más de una hora? *

Marca solo un óvalo.

- a. Sí, me limita mucho
- b. Sí, me limita un poco
- c. No, no me limita nada

57. 5) Su salud actual, ¿le limita para coger o llevar la bolsa de la compra? *

Marca solo un óvalo.

- a. Sí, me limita mucho
- b. Sí, me limita un poco
- c. No, no me limita nada

58. 6) Su salud actual, ¿le limita para subir varios pisos por la escalera? *

Marca solo un óvalo.

- a. Sí, me limita mucho
- b. Sí, me limita un poco
- c. No, no me limita nada

59. 7) Su salud actual, ¿le limita para subir un solo piso por la escalera? *

Marca solo un óvalo.

- a. Sí, me limita mucho
- b. Sí, me limita un poco
- c. No, no me limita nada

60. 8) Su salud actual, ¿le limita para agacharse o arrodillarse? *

Marca solo un óvalo.

- a. Sí, me limita mucho
- b. Sí, me limita un poco
- c. No, no me limita nada

61. 9) Su salud actual, ¿le limita para caminar un kilómetro o más? *

Marca solo un óvalo.

- a. Sí, me limita mucho
- b. Sí, me limita un poco
- c. No, no me limita nada

62. 10) Su salud actual, ¿le limita para caminar varios centenares de metros (varias manzanas)? *

Marca solo un óvalo.

- a. Sí, me limita mucho
- b. Sí, me limita un poco
- c. No, no me limita nada

63. 11) Su salud actual, ¿le limita para caminar unos 100 metros (una sola manzana)? *

Marca solo un óvalo.

- a. Sí, me limita mucho
- b. Sí, me limita un poco
- c. No, no me limita nada

64. 12) Su salud actual, ¿le limita para bañarse o vestirse por sí mismo? *

Marca solo un óvalo.

- a. Sí, me limita mucho
- b. Sí, me limita un poco
- c. No, no me limita nada

Las siguientes preguntas se refieren a problemas en su trabajo o en sus actividades diarias a causa de su salud física

65. 13) Durante las últimas 4 semanas, ¿tuvo que reducir el tiempo dedicado al trabajo o a sus actividades cotidianas? *

Marca solo un óvalo.

- a. Sí
- b. No

66. 14) Durante las últimas 4 semanas, ¿hizo menos de lo que hubiera querido hacer? *

Marca solo un óvalo.

- a. Sí
- b. No

67. 15) Durante las últimas 4 semanas, ¿tuvo que dejar de hacer algunas tareas en su trabajo o en sus actividades cotidianas? *

Marca solo un óvalo.

- a. Sí
- b. No

68. 16) Durante las últimas 4 semanas, ¿tuvo dificultad para hacer su trabajo o sus actividades cotidianas (por ejemplo, le costó más de lo normal)? *

Marca solo un óvalo.

- a. Sí
- b. No

69. 17) Durante las últimas 4 semanas, ¿tuvo que reducir el tiempo dedicado al trabajo o a sus actividades cotidianas a causa de algún problema emocional (como estar triste, deprimido, o nervioso)? *

Marca solo un óvalo.

- a. Sí
- b. No

70. 18) Durante las últimas 4 semanas, ¿hizo menos de lo que hubiera querido hacer a causa de algún problema emocional (como estar triste, deprimido, o nervioso)? *

Marca solo un óvalo.

- a. Sí
- b. No

71. 19) Durante las últimas 4 semanas, ¿no hizo su trabajo o sus actividades cotidianas tan cuidadosamente como de costumbre a causa de algún problema emocional (como estar triste, deprimido, o nervioso)? *

Marca solo un óvalo.

- a. Sí
- b. No

72. 20) Durante las últimas 4 semanas, ¿hasta qué punto su salud física o los problemas emocionales han dificultado sus actividades sociales habituales con la familia, los amigos, los vecinos u otras personas? *

Marca solo un óvalo.

- a. Nada
- b. Un poco
- c. Regular
- d. Bastante
- e. Mucho

73. 21) ¿Tuvo dolor en alguna parte del cuerpo durante las 4 últimas semanas? *

Marca solo un óvalo.

- a. No, ninguno
- b. Sí, muy poco
- c. Sí, un poco
- d. Sí, moderado
- e. Sí, mucho
- f. Sí, muchísimo

74. 22) Durante las últimas 4 semanas, ¿hasta qué punto el dolor le ha dificultado su trabajo habitual (incluido el trabajo fuera de casa y las tareas domésticas)? *

Marca solo un óvalo.

- a. Nada
- b. Un poco
- c. Regular
- d. Bastante
- e. Mucho

Las siguientes preguntas se refieren a cómo se ha sentido y cómo le han ido las cosas durante las 4 últimas semanas. En cada pregunta, responda lo que se parezca más a cómo se ha sentido usted.

75. 23) Durante las 4 últimas semanas, ¿con qué frecuencia se sintió lleno de vitalidad? *

Marca solo un óvalo.

- a. Siempre
- b. Casi siempre
- c. Muchas veces
- d. Algunas veces
- e. Sólo alguna vez
- f. Nunca

76. 24) Durante las 4 últimas semanas, ¿con qué frecuencia estuvo muy nervioso? *

Marca solo un óvalo.

- a. Siempre
- b. Casi siempre
- c. Muchas veces
- d. Algunas veces
- e. Sólo alguna vez
- f. Nunca

77. 25) Durante las 4 últimas semanas, ¿con qué frecuencia se sintió tan bajo de moral que nada podía animarle? *

Marca solo un óvalo.

- a. Siempre
- b. Casi siempre
- c. Muchas veces
- d. Algunas veces
- e. Sólo alguna vez
- f. Nunca

78. 26) Durante las 4 últimas semanas, ¿con qué frecuencia se sintió calmado y tranquilo? *

Marca solo un óvalo.

- a. Siempre
- b. Casi siempre
- c. Muchas veces
- d. Algunas veces
- e. Sólo alguna vez
- f. Nunca

79. 27) Durante las 4 últimas semanas, ¿con qué frecuencia tuvo mucha energía? *

Marca solo un óvalo.

- a. Siempre
- b. Casi siempre
- c. Muchas veces
- d. Algunas veces
- e. Sólo alguna vez
- f. Nunca

80. 28) Durante las 4 últimas semanas, ¿con qué frecuencia se sintió desanimado y triste? *

Marca solo un óvalo.

- a. Siempre
- b. Casi siempre
- c. Muchas veces
- d. Algunas veces
- e. Sólo alguna vez
- f. Nunca

81. 29) Durante las 4 últimas semanas, ¿con qué frecuencia se sintió agotado? *

Marca solo un óvalo.

- a. Siempre
- b. Casi siempre
- c. Muchas veces
- d. Algunas veces
- e. Sólo alguna vez
- f. Nunca

82. 30) Durante las 4 últimas semanas, ¿con qué frecuencia se sintió feliz? *

Marca solo un óvalo.

- a. Siempre
- b. Casi siempre
- c. Muchas veces
- d. Algunas veces
- e. Sólo alguna vez
- f. Nunca

83. 31) Durante las 4 últimas semanas, ¿con qué frecuencia se sintió cansado? *

Marca solo un óvalo.

- a. Siempre
- b. Casi siempre
- c. Muchas veces
- d. Algunas veces
- e. Sólo alguna vez
- f. Nunca

84. 32) Durante las 4 últimas semanas, ¿con qué frecuencia la salud física o los problemas emocionales le han dificultado sus actividades sociales (como visitar a amigos o familiares)? *

Marca solo un óvalo.

- a. Siempre
- b. Casi siempre
- c. Algunas veces
- d. Sólo alguna vez
- e. Nunca

Por favor, diga si le parece CIERTA o FALSA cada una de las siguientes frases

85. 33) Creo que me pongo enfermo más fácilmente que otras personas *

Marca solo un óvalo.

- a. Totalmente cierta
- b. Bastante cierta
- c. No lo sé
- d. Bastante falsa
- e. Totalmente falsa

86. 34) Estoy tan sano como cualquiera *

Marca solo un óvalo.

- a. Totalmente cierta
- b. Bastante cierta
- c. No lo sé
- d. Bastante falsa
- e. Totalmente falsa

87. 35) Creo que mi salud va a empeorar *

Marca solo un óvalo.

- a. Totalmente cierta
- b. Bastante cierta
- c. No lo sé
- d. Bastante falsa
- e. Totalmente falsa

88. 36) Mi salud es excelente *

Marca solo un óvalo.

- a. Totalmente cierta
- b. Bastante cierta
- c. No lo sé
- d. Bastante falsa
- e. Totalmente falsa

89. Indique de nuevo si es paciente de fibromialgia para dirigirla a unas preguntas específicas sobre la enfermedad: *

Marca solo un óvalo.

- Sí
- No *Salta a la pregunta 111*

Cuestionario de Impacto de la Fibromialgia (FIQ-R)

1- INSTRUCCIONES: Para cada pregunta marque una X en la casilla que mejor indique la dificultad con la que ha realizado cada una de las siguientes actividades a causa de la fibromialgia durante la última semana:

90. a) Peinarse *

Marca solo un óvalo.

	0	1	2	3	4	5	6	7	8	9	10	
Ninç												Máxima

91. b) Caminar 20 minutos sin necesidad de pararse *

Marca solo un óvalo.

	0	1	2	3	4	5	6	7	8	9	10	
Ninç												Máxima

92. c) Preparar la comida *

Marca solo un óvalo.

	0	1	2	3	4	5	6	7	8	9	10	
Ninç												Máxima

93. d) Barrer, fregar o pasar la aspiradora *

Marca solo un óvalo.

	0	1	2	3	4	5	6	7	8	9	10	
Ning	◯	◯	◯	◯	◯	◯	◯	◯	◯	◯	◯	Máxima

94. e) Levantar y transportar una bolsa de la compra llena *

Marca solo un óvalo.

	0	1	2	3	4	5	6	7	8	9	10	
Ning	◯	◯	◯	◯	◯	◯	◯	◯	◯	◯	◯	Máxima

95. f) Subir escaleras *

Marca solo un óvalo.

	0	1	2	3	4	5	6	7	8	9	10	
Ning	◯	◯	◯	◯	◯	◯	◯	◯	◯	◯	◯	Máxima

96. g) Cambiar la ropa de la cama *

Marca solo un óvalo.

	0	1	2	3	4	5	6	7	8	9	10	
Ning	◯	◯	◯	◯	◯	◯	◯	◯	◯	◯	◯	Máxima

97. h) Estar sentada en una silla durante 45 minutos *

Marca solo un óvalo.

	0	1	2	3	4	5	6	7	8	9	10	
Ning	◯	◯	◯	◯	◯	◯	◯	◯	◯	◯	◯	Máxima

98. i) Hacer la compra *

Marca solo un óvalo.

	0	1	2	3	4	5	6	7	8	9	10	
Ning	◯	◯	◯	◯	◯	◯	◯	◯	◯	◯	◯	Máxima

2- INSTRUCCIONES: Para cada pregunta marque una X en la casilla que mejor indique la influencia global que ha ejercido su fibromialgia en los últimos 7 días

99. a) La fibromialgia me impidió hacer lo que tenía proyectado esta semana *

Marca solo un óvalo.

	0	1	2	3	4	5	6	7	8	9	10	
Nun	◯	◯	◯	◯	◯	◯	◯	◯	◯	◯	◯	Siempre

100. b) Los síntomas de mi fibromialgia me tuvieron totalmente abrumada *

Marca solo un óvalo.

	0	1	2	3	4	5	6	7	8	9	10	
Nun	◯	◯	◯	◯	◯	◯	◯	◯	◯	◯	◯	Siempre

3- INSTRUCCIONES: Para cada pregunta marque una X en la casilla que mejor indique la intensidad de los síntomas de su fibromialgia durante los últimos 7 días

101. a) Dolor *

Marca solo un óvalo.

	0	1	2	3	4	5	6	7	8	9	10	
Ninç												Dolor insoportable

102. b) Energía *

Marca solo un óvalo.

	0	1	2	3	4	5	6	7	8	9	10	
Muc												Ninguna energía

103. c) Rigidez *

Marca solo un óvalo.

	0	1	2	3	4	5	6	7	8	9	10	
Ninç												Rigidez intensa

104. d) Calidad del sueño *

Marca solo un óvalo.

	0	1	2	3	4	5	6	7	8	9	10	
Me l												Me levanto muy cansada

105. e) Depresión *

Marca solo un óvalo.

	0	1	2	3	4	5	6	7	8	9	10	
Nad	◯	◯	◯	◯	◯	◯	◯	◯	◯	◯	◯	Muy deprimida

106. f) Problemas de memoria *

Marca solo un óvalo.

	0	1	2	3	4	5	6	7	8	9	10	
Bue	◯	◯	◯	◯	◯	◯	◯	◯	◯	◯	◯	Muy mala memoria

107. g) Ansiedad *

Marca solo un óvalo.

	0	1	2	3	4	5	6	7	8	9	10	
Nad	◯	◯	◯	◯	◯	◯	◯	◯	◯	◯	◯	Muy ansiosa

108. h) Dolorimiento al tacto *

Marca solo un óvalo.

	0	1	2	3	4	5	6	7	8	9	10	
Ninç	◯	◯	◯	◯	◯	◯	◯	◯	◯	◯	◯	Muchísimo

109. i) Problemas de equilibrio *

Marca solo un óvalo.

	0	1	2	3	4	5	6	7	8	9	10	
Ning	◯	◯	◯	◯	◯	◯	◯	◯	◯	◯	◯	Severos

110. j) Grado de sensibilidad al ruido intenso, la luz brillante, los olores, el frío *

Marca solo un óvalo.

	0	1	2	3	4	5	6	7	8	9	10	
Ning	◯	◯	◯	◯	◯	◯	◯	◯	◯	◯	◯	Mucha sensibilidad

Cuestionario de síntomas

Marque el nivel que mejor describa cada síntoma en su caso:
0: Ausente
1: Ligero
2: Medio
3: Severo

111. Dolor abdominal/epigástrico *

Marca solo un óvalo.

	0	1	2	3	
Aus	◯	◯	◯	◯	Severo

112. Hinchazón *

Marca solo un óvalo.

	0	1	2	3	
Aus	◯	◯	◯	◯	Severo

113. Gases *

Marca solo un óvalo.

	0	1	2	3	
Aus	◯	◯	◯	◯	Severo

114. Diarrea *

Marca solo un óvalo.

	0	1	2	3	
Aus	◯	◯	◯	◯	Severo

115. Estreñimiento *

Marca solo un óvalo.

	0	1	2	3	
Aus	◯	◯	◯	◯	Severo

116. Urgencia para defecar *

Marca solo un óvalo.

	0	1	2	3	
Aus	◯	◯	◯	◯	Severo

117. Evacuación incompleta *

Marca solo un óvalo.

	0	1	2	3	
Aus	○	○	○	○	Severo

118. Naúseas/vómitos *

Marca solo un óvalo.

	0	1	2	3	
Aus	○	○	○	○	Severo

119. Ardor/acidez *

Marca solo un óvalo.

	0	1	2	3	
Aus	○	○	○	○	Severo

120. Piel seca/picores/granos *

Marca solo un óvalo.

	0	1	2	3	
Aus	○	○	○	○	Severo

121. Quemazón de la piel *

Marca solo un óvalo.

	0	1	2	3	
Aus	◯	◯	◯	◯	Severo

122. Pitido en los oídos (acufeno) *

Marca solo un óvalo.

	0	1	2	3	
Aus	◯	◯	◯	◯	Severo

123. Hormigueo en extremidades *

Marca solo un óvalo.

	0	1	2	3	
Aus	◯	◯	◯	◯	Severo

124. Migraña *

Marca solo un óvalo.

	0	1	2	3	
Aus	◯	◯	◯	◯	Severo

Cuestionario de adherencia a la dieta mediterránea - PREDIMED

125. ¿Usa usted el aceite de oliva como principal grasa para cocinar? *

Marca solo un óvalo.

- Sí
- No

126. ¿Cuánto aceite de oliva consume en total al día (incluyendo el usado para freír, el de las comidas fuera de casa, las ensaladas, etc.)? *

Marca solo un óvalo.

- Más de 2 cucharadas
- Menos de 2 cucharadas

127. ¿Cuántas raciones de verdura u hortalizas consume al día? (las guarniciones o acompañamientos contabilizan como 1/2 ración) *

Marca solo un óvalo.

- Más de 2 al día (al menos una de ellas en ensaladas o crudas)
- Menos de 2 al día

128. ¿Cuántas piezas de fruta (incluyendo zumo natural) consume al día? *

Por ejemplo, 1 manzana, 10-12 cerezas, 1 pera, etc.

Marca solo un óvalo.

- Más de 3 al día
- Menos de 3 al día

129. ¿Cuántas raciones de carnes rojas, hamburguesas, salchichas o embutidos consume al día (una ración equivale a 100-150 gr)? *

Marca solo un óvalo.

- Más de 1 al día
- Menos de 1 al día

130. ¿Cuántas raciones de mantequilla, margarina o nata consume al día (una porción individual equivale a 12 gr)? *

Marca solo un óvalo.

- Más de 1 al día
- Menos de 1 al día

131. ¿Cuántas bebidas con o sin gas y/o azucaradas (refrescos, colas, tónicas, otras) consume al día? *

Una ración equivale a un vaso estándar de aproximadamente 250ml.

Marca solo un óvalo.

- Más de 1 al día
- Menos de 1 al día

132. ¿Bebe vino? ¿Cuánto consume a la semana? *

Marca solo un óvalo.

- Más de 3 vasos a la semana
- Menos de 3 vasos a la semana

133. ¿Cuántas raciones de legumbres consume a la semana (una ración o plato equivale a 150 gr)? *

Marca solo un óvalo.

- Más de 3 a la semana
- Menos de 3 a la semana

134. ¿Cuántas raciones de pescado o mariscos consume a la semana (un plato, pieza o ración equivale a 100-150 gr de pescado ó 4-5 piezas de marisco)? *

Marca solo un óvalo.

- Más de 3 a la semana
- Menos de 3 a la semana

135. ¿Cuántas veces consume repostería comercial (no casera) como galletas, flanes, dulces o pasteles a la semana? *

Marca solo un óvalo.

- Más de 3 a la semana
- Menos de 3 a la semana

136. ¿Cuántas veces consume frutos secos a la semana (una ración equivale a 30 gr)? *

Una ración de 30 gramos equivale a un puñado mediano-grande. Ejemplo: 10-15 almendras o 5 nueces.

Marca solo un óvalo.

- Más de 1 a la semana
- Menos de 1 a la semana

137. ¿Cuántas veces consume preferentemente carne de pollo, pavo o conejo en vez de ternera, cerdo, hamburguesas o salchichas? *

Ejemplo: una pieza o ración de carne de pollo equivale a 100-150 gr

Marca solo un óvalo.

- Más de 1 a la semana
- Menos de 1 a la semana

138. ¿Cuántas veces a la semana consume los vegetales cocinados, la pasta, el arroz u otros platos aderezados con una salsa de tomate, ajo, cebolla o puerro elaborada a fuego lento con aceite de oliva (sofrito)? *

Marca solo un óvalo.

- Más de 2 a la semana
- Menos de 2 a la semana

139. ¿Cuánto alcohol bebe de forma habitual? (tipo de bebida, frecuencia y cantidad) *

Ejemplo: 2 latas de cerveza, 1 vermut y 1 copa de vino blanco a la semana

140. Indique otras observaciones de interés que deberíamos tener en cuenta sobre su dieta *

Por último... Estudio de prevalencia de endometriosis en FM

El objetivo es evaluar la probable mayor prevalencia de la endometriosis en las pacientes diagnosticadas de FM.

141. ¿Tiene hijos? *

Marca solo un óvalo.

- Sí *Salta a la pregunta 143*
- No *Salta a la pregunta 142*

142. ¿Ha intentado tener hijos y no ha tenido éxito? *

Marca solo un óvalo.

- Sí
- No

143. Edad de la primera regla *

____

144. ¿Ha sufrido abortos o embarazos ectópicos? *

Marca solo un óvalo.

- Sí
- No

145. ¿Es posible que tenga heridas quirúrgicas debidas a cesárea o episiotomía u otra intervención ginecológica? *

Marca solo un óvalo.

- Sí
- No

146. ¿Padece infertilidad asociada a algún factor? *

Marca solo un óvalo.

- Sí
- No

147. ¿Tiene diagnóstico de endometriosis? *

Marca solo un óvalo.

- Sí
- No

148. ¿Sufre periodos menstruales abundantes y dolorosos? *

Marca solo un óvalo.

- Sí
- No

149. ¿Sufre dolores pélvicos? *

Marca solo un óvalo.

- Sí
- No

150. ¿Sufre ovario poliquístico? *

Marca solo un óvalo.

- Sí
- No

Cuestionario general

151. Durante las 4 últimas semanas, ¿Le resulta/ha resultado difícil caminar por el dolor? *

Marca solo un óvalo.

- Nunca
- Rara vez
- En ocasiones
- Frecuentemente
- Siempre

152. Durante las 4 últimas semanas, ¿Siente que los síntomas abdominales y dificultad al caminar le impiden hacer vida normal? *

Marca solo un óvalo.

- Nunca
- Rara vez
- En ocasiones
- Frecuentemente
- Siempre

153. Durante las 4 últimas semanas, ¿Tiene/tuvo cambios de humor bruscos? *

Marca solo un óvalo.

- Nunca
- Rara vez
- En ocasiones
- Frecuentemente
- Siempre

154. Durante las 4 últimas semanas, ¿Ha sentido que los demás no entienden lo que le está pasando? *

Marca solo un óvalo.

- Nunca
- Rara vez
- En ocasiones
- Frecuentemente
- Siempre

155. Durante las 4 últimas semanas, ¿Ha sentido que su apariencia haya cambiado? *

Marca solo un óvalo.

- Nunca
- Rara vez
- En ocasiones
- Frecuentemente
- Siempre

156. Indique otras observaciones de interés que deberíamos tener en cuenta sobre su salud general y la enfermedad, si la padece: *

Este contenido no ha sido creado ni aprobado por Google.

Google Formularios
